# The Impacts of Work-Life Balance on the Emotional Exhaustion and Well-Being of College Teachers in China

**DOI:** 10.3390/healthcare10112234

**Published:** 2022-11-08

**Authors:** Changwu Wei, Jian-Hong Ye

**Affiliations:** 1 Dhurakij Pundit University, Bangkok 10900, Thailand; 2 College of Education and Music, Hezhou University, Hezhou 542899, China; 3 Faculty of Education, Beijing Normal University, Beijing 100875, China

**Keywords:** college teachers, emotional exhaustion, sustainable development, well-being, work-life balance

## Abstract

UN Sustainable Development Goal 3 states that “Ensuring healthy lives and promoting well-being at all ages is essential to sustainable development.” The sustainable well-being of college teachers deserves attention. Currently, college teachers in China are facing enormous challenges and pressures, which may increase their emotional exhaustion (EE) and hinder the sustainable development of their well-being (WB). Therefore, this study examined Chinese college teachers’ well-being in relation to their work-life balance (WLB) and EE. An online survey was conducted. The valid subjects included 586 college teachers (367 females, 62.6%). We assessed their WLB, EE, and WB. The results revealed that among the Chinese college teachers, (1) WLB was negatively correlated with EE, but positively correlated with WB; EE was negatively correlated with WB; (2) EE partially mediated the relationship between WLB and WB; and (3) College teachers who are male, class tutors, and in public colleges scored higher on EE and lower on WLB and WB than those who are female, non-class tutors, and in private colleges, respectively. The findings indicated that WLB is an important factor for the sustainable development of the well-being of college teachers, and special attention should be paid to college teachers who are male, class tutors, and in public colleges in China.

## 1. Introduction

Based on the Millennium Development Goals, the United Nations proposed the 2030 Sustainable Development Goals, the third of which is to “ensure healthy lives and promote well-being for all at all ages” [1]. In sustainable development, universities, as change agents, shape their own way of sustainable growth by offering knowledge, skills, and employment opportunities [2], and play a significant part in helping foster community development and creating a sustainable future in communities [3,4]. According to UNESCO [5], teachers, as the essential driving force, facilitate the reform of the educational process and the sustainable development of educational institutions. In this sense, the well-being of college teachers helps to ensure the sustainability of this driving force. However, college teachers often face great pressure and experience a high level of burnout owing to the challenges of their jobs [6]. They also report lower levels of well-being than other professions [7]. The COVID-19 pandemic has put forward new job demands and challenges for college teachers, which has a negative impact on the well-being of college teachers [8,9]. With the connotative development and transformation of Chinese education, college teachers shoulder arduous missions and expectations, which bring them great challenges and pressures, and may aggravate their emotional exhaustion (EE) [10,11]. Chinese college teachers also faced new changes and challenges during the COVID-19 pandemic [12], thus impeding the sustainable development of their well-being (WB).

College teachers face continuous work-related tension and pressure, including excessive workload, curriculum quality requirements, performance evaluation, various urgent tasks, scientific research, and frequent academic activities, as well as other work-related activities [13,14,15]. In addition, they play multiple roles, such as parents, husbands, or wives in their personal lives, researchers or employees in their professional lives, and students in their career development [16]. Maintaining a work-life balance (WLB) is a challenge for college teachers due to the job responsibilities and factors that aggravate their stress [17]. The impact of the COVID-19 pandemic has made it more difficult for college teachers to maintain a healthy work-life balance [18], especially for college teachers who are mothers [19,20], which in turn affects their well-being [21]. Compared with other positions in higher education institutions, college teachers have the most precarious WLB [22,23]. According to the literature review of Franco et al. [24], studies on college teachers’ WLB and WB are not sufficient, and the association between WLB and WB remains unclear. To cope with the stress caused by WLB, it is necessary to explore college teachers’ WB in relation to their WLB and to explore ways to promote both their WLB and WB. 

Currently, college teachers’ working hours are constantly increasing [25]. One of the important features of the teachers’ profession is work-related stress [26,27], which has put college teachers under increasing pressure [28], and has made them vulnerable to EE [29]. College teachers often experience EE due to work overload [30]. Some studies on Chinese college teachers have also found that those with high work pressure experience more EE [31,32]. The EE of college teachers will affect their physical health [33] and their mental health [34], thus leading to a low level of WB [35]. To cope with the negative emotions caused by work pressure, there is a call for studies on college teachers’ WB in relation to EE, in the hope of fostering their ability to overcome EE and seek WB. However, existing research on EE and WB has focused on teachers in elementary schools, secondary schools, and high schools [36,37]; however, college teachers face different challenges [13,14,15]. Therefore, there is a need for more empirical studies on the effect of EE on college teachers’ WB. In addition, few studies have explored EE as a mediator between WLB and WB, especially among college teachers. 

Moreover, research on gender differences in WLB, WB, and EE among college teachers is inconsistent. Some studies have found that male teachers had stronger perceptions of WLB than female teachers [38,39,40], while other studies found that male teachers’ perceptions of WLB were weaker than those of female teachers [41], and yet others found no gender difference [42,43]. Similarly, some studies have shown that women have stronger perceptions of WB than men [44,45], but another study found no gender difference [46]. As for the perception of EE, studies have found no gender difference among college teachers [47,48]. Likewise, the results of differences in school type (public vs. private) are also inconsistent. For example, studies have suggested that public and private college teachers have no significant difference in their perceptions of WLB [38,40]. However, some studies have suggested that teachers in private colleges work in poorer working conditions, have fewer opportunities for mid-career studies, research and training, and even feel less respect from students; therefore, they may have weaker perceptions of WLB than teachers in public colleges [45]. In terms of WB and EE, the perceptions of teachers in public colleges are stronger than those of teachers in private colleges [46], and the perceptions of EE of teachers in private colleges are stronger than those of teachers in public colleges [49]. In particular, serving as class tutors is an inevitable experience for many teachers in Chinese colleges [50]. In addition to teaching, performing scientific research and serving society, they also need to guide students to study and perform self-management, and carry out mental health education [51]. They are faced with more responsibilities and difficulties than college teachers who are non-class tutors [52]. Therefore, the perceptions of EE of college teachers who concurrently serve as class tutors are significantly stronger than those of non-class tutors [53]. However, as far as we know, there is no research on WLB and WB of class tutors among college teachers in China. 

Therefore, a cross-sectional research design was adopted to explore Chinese college teachers’ WB in relation to their WLB and EE, and to examine the mediating role of EE in the association between WLB and WB. We also aimed to explore the characteristics of college teachers’ WLB, EE, and WB in terms of gender, college type, and the roles of college teachers.

## 2. Hypothesis Development

### 2.1. Work-Life Balance and Well-Being

According to Wayne et al. [54], WLB is the harmony of work and non-work roles. Work roles refer to the role engaged in for the purpose of getting paid, and non-work roles refer to other roles besides work roles, such as family, leisure, personal growth, etc., [55]. The term work-life balance has been used interchangeably with role balance, work-family balance, and work-non-work balance [55]; work-life balance was used in this study. Terms such as WB, happiness, subjective WB, and psychological quality of life are often used interchangeably in related studies [56]. In the present study, we consider WB to be a positive psychological experience, such as happiness, joy, and other pleasant experiences, arising from satisfying personal needs and realizing personal expectations [56]. 

It has been demonstrated that WLB is a major factor linked to WB [24]. After controlling socioeconomic factors, a good WLB for laborers in Pakistan was found to have a positive and significant relationship with their WB [57]. In addition, WLB has been found to be positively correlated with WB in studies on rural women in Sichuan, China [58], teachers in the United Arab Emirates [59], and hospice nurses [60]. Therefore, the following hypothesis was proposed:

**Hypothesis** **1** **(H1).** *WLB is positively correlated with WB among Chinese college teachers.*

### 2.2. Work-Life Balance and Emotional Exhaustion

EE is a stress response caused by excessive mental exertion and extreme fatigue under pressure [61]. Studies have demonstrated that WLB helps alleviate EE [62,63,64], and one of the consequences of work-life imbalance is EE [65]. For example, those who experienced more difficulties in WLB reported higher EE [66,67]. That is, increased work stress results in work-life imbalance, which in turn leads to EE [68]. Mahendran’s study also confirmed a positive correlation between work–family imbalance and EE [69]. Based on the studies above, we believed that WLB may be negatively correlated with EE among college teachers in China. Therefore, the following hypothesis was proposed in this study:

**Hypothesis** **2** **(H2).** *WLB is negatively correlated with EE among Chinese college teachers.*

### 2.3. Emotional Exhaustion and Well-Being

According to Salas-Vallina et al., the lack of EE is a dimension of WB [70]. Studies on EE and WB have mainly focused on medical and health personnel. For example, as a result of treating psychological problems, psychiatrists are prone to EE, which affects their general WB [37]. Arrogante and Aparicio-Zaldivar found that high scores for EE predict low levels of WB among intensive care professionals [36]. Alhadi et al. argued that there is a need for the improvement of mental health professionals’ WB, as they are at a high risk of EE [71]. In addition, some studies on college teachers have also revealed that EE is negatively associated with WB [72]. Therefore, we proposed the following hypothesis:

**Hypothesis** **3** **(H3).** *EE is negatively correlated with WB among Chinese college teachers.*

### 2.4. Work-Life Balance, Emotional Exhaustion, and Well-Being

Neumann et al. argued that teachers who reported EE were more likely to experience work-life imbalance and suffer low levels of WB [73]. This finding suggests that EE may play a certain role between the other two variables. Studies have demonstrated that WLB is positively correlated with WB [57,58] and negatively correlated with EE [63,67], which is a negative factor related to WB [71,74]. It follows that EE may mediate the relationship between WLB and WB. In addition, according to the JDR model [75], job resources promote favorable job outcomes and reduce job burnout, while job burnout hinders favorable job outcomes. Alternatively, job resources facilitate job goals by decreasing job burnout; that is, job burnout plays a mediating role in the association between job resources and job goals. In the current study, WLB was considered as a job resource, WB as a favorable job outcome, and EE as one indicator of job burnout. Therefore, the following hypothesis was proposed:

**Hypothesis** **4** **(H4).** *EE mediates the association between WLB and WB among Chinese college teachers.*

**Figure 1:**
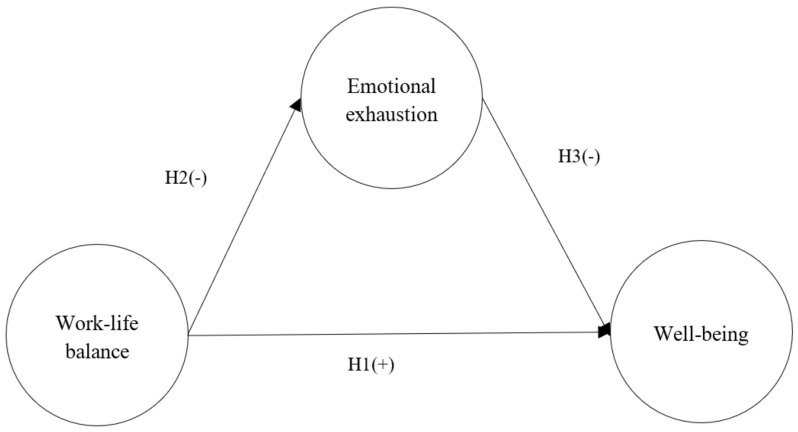
Research Model.

Overall, the main aim of the study was to examine the impacts of WLB on EE and WB, and the mediating role of EE in the relationship between WLB and WB. The concept framework of this study is presented in Figure 1.

## 3. Materials and Methods

### 3.1. Procedures and Participants 

Due to the COVID-19 pandemic, it is challenging to recruit a sufficient number of research samples, so snowball sampling may be an effective sampling technique to recruit participants [76,77]. Therefore, in this study, we distributed the questionnaire link and QR code to college teachers whom the authors knew through social media. When they completed the questionnaire anonymously, they sent the link or QR code to other college teachers they knew. Details of the research purpose, data filling object, data use, and anonymity of information were presented in the questionnaire, and the participants gave informed consent by completing and submitting the questionnaire. The data were collected from 8 February to 30 March 2022. A total of 600 questionnaires were returned, of which 586 were considered valid (giving an effective rate of 97.7%), after 14 were excluded because the answers were too regular (i.e., the same answers for all items) or they chose paradoxical options. 

The sample, therefore, consisted of 586 college teachers who were from the vast majority of provinces and cities (28 provinces or autonomous regions) in mainland China, and the samples were representative to a certain extent. The entire sample included 367 females (62.6%) and 219 males (37.4%); 383 were class tutors (65.4%) and 203 were not (34.6%); and 361 were from public colleges (61.6%), while the remaining 225 were from private colleges (38.4%).

### 3.2. Measures

The questionnaire comprised scales measuring WLB, EE, and WB. Based on the literature, we developed items for the questionnaire which adopted a 5-point Likert scale, with 1 for strongly disagree to 5 for strongly agree. 

In the present study, the concept of WLB was consistent with Wayne et al.’s work–nonwork balance; therefore, the five-item original Global Balance Scale developed by Wayne et al. [54] was chosen to investigate the global WLB of college teachers in China. An example item is, “Overall, my work and nonwork roles are integrated.” In this study, Cronbach’s α was 0.92.

The Maslach Burnout Inventory—General Survey (Chinese Version) (MBI—GSC) revised by Li has been widely used in China [61]. The five-item version emotional exhaustion subscale was adopted in this study to measure Chinese college teachers’ EE. In this study, Cronbach’s α was 0.90.

WB was measured using the Chinese Happiness Scale-Short Version (CHSSV) revised by Nong et al., which has good reliability and validity and is suitable for Chinese samples [78]. The CHSSV contains 10 items. Sample items are “I feel happy” and “I have a sense of accomplishment in life.” In this study, Cronbach’s α was 0.87.

### 3.3. Data Analysis Strategies

Employing AMOS 23.0 and SPSS 26.0 for statistical analyses, this study used a structural equation model (SEM) to evaluate the hypothesis framework. Before the data analysis, 14 copies were excluded because the answers were too regular (the same answers for all items) or they chose paradoxical options. After Harman’s one-factor test for common method variance (CMV) was run on the variables [79], confirmatory factor analysis (CFA) was performed for the measurement model [80] as the CMV was not serious. Then, the reliability and validity were checked according to the criteria suggested by Hair et al. [81], and an analysis of variance was performed [82]. Student’s *t*-test is commonly used to compare the differences between the mean values of two groups [83], so was used in this study. The bootstrap method was also used to examine EE as a mediator between WLB and WB [84]. 

#### 3.3.1. Common Method Variance

When adopting an online questionnaire, to limit CMV [85] Harman’s one-factor test can be run to test the CMV of the study variables [79]. In this study, we performed exploratory factor analysis for all 20 items in the study scales, and then tested the results of non-rotated factor analysis. The first factor showed 43% of the explanatory power (threshold value: 50%), which indicated that the CMV of the variables in this study was not serious [79]. 

#### 3.3.2. Measurement Model Fit Indexes

This study used first-order CFA to test the model fit indexes of the measured variables. The χ^2^/*df* value should be less than 5, GFI and AGFI should be greater than 0.80, and RMSEA should be less than 0.10 [80]. As shown in Table 1, the χ^2^/*df* value of the WB construct was a little more than 5, GFI and AGFI were higher than 0.80, and RMSEA was less than 0.10, indicating an acceptable model.

#### 3.3.3. Reliability and Validity Analysis

The Cronbach’s α and CR value should be at least 0.70 [81]. The Cronbach’s α values of all scales in this study ranged from 0.87 to 0.92, and the CR values from 0.90 to 0.93, indicating that the internal consistency reliability was acceptable, as shown in Table 2. 

According to the criteria for convergence validity, the acceptable factor loading value should be at least 0.50, and the average variance extracted (AVE) value should be at least 0.50 [81]. The factor loading (FL) values of the dimensions in this study ranged from 0.51 to 0.92, and the AVE values ranged from 0.58 to 0.69, as shown in Table 2, indicating that convergence validity for each dimension was acceptable.

The AVE square root of the dimension should be greater than its correlation coefficients with other dimensions [86]. The square root of the AVE for each dimension was between 0.76 and 0.83, which was greater than the correlation coefficients of each dimension, as shown in Table 3, indicating that discriminant validity for each dimension was acceptable.

## 4. Results

### 4.1. Chinese College Teachers’ Perceptions of WLB, EE, and WB

As can be seen from Table 4, male teachers had significantly weaker perceptions of WLB than female teachers (*t* = −2.76, *p* < 0.01); class tutors had significantly weaker perceptions of WLB than non-class tutors (*t* = −7.33, *p* < 0.001); and the public college teachers’ perceptions of WLB were weaker than those of private college teachers (*t* = −2.01, *p* < 0.05). In terms of EE, male teachers had stronger perceptions than female teachers (*t* = 2.99, *p* < 0.01); class tutors had significantly stronger perceptions than non-class tutors (*t* = 9.18, *p* < 0.001); and teachers’ perceptions in public colleges were stronger than those of teachers in private colleges (*t* = 2.89, *p* < 0.01). As for WB, male teachers had significantly weaker perceptions than female teachers (*t* = −2.13, *p* < 0.05); class tutors had significantly weaker perceptions than non-class tutors (*t* = −7.93, *p* < 0.001); and the perceptions of WB among public college teachers were weaker than those of private college teachers (*t* = −3.04, *p* < 0.01).

### 4.2. Model Fit Analysis

AMOS 23.0 was used to check the fitness of the research model. RMSEA should be lower than 0.10, GFI, AGFI, NFI, and NNFI not less than 0.90, CFI, IFI, and RFI not less than 0.80, and PNFI and PGFI not less than 0.50 [80] (578–581). Fit index values of this study were as follows: RMSEA = 0.06, GFI = 0.91, AGFI = 0.89, NFI = 0.95, NNFI = 0.96, CFI = 0.96, IFI = 0.96, RFI = 0.94, PNFI = 0.83, and PGFI = 0.73. Except for AGFI = 0.89 (which was very close to the criterion of 0.9), all the other indicators are in line with the criteria, indicating that the model was acceptable.

### 4.3. Direct Effects Analysis

**Figure 2:**
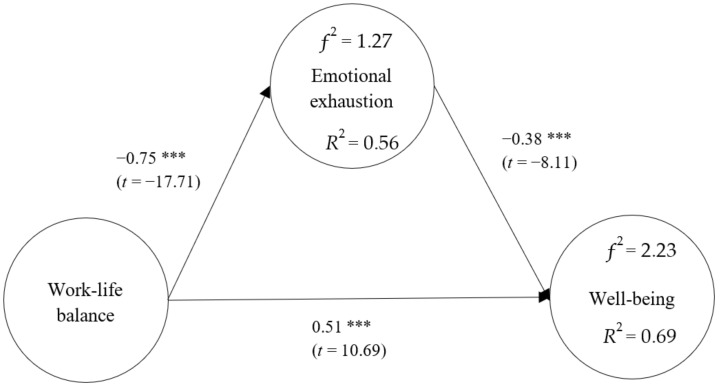
Verification of the Research Model. ***p < 0.001.

The direct effect among the variables was tested using the bootstrapping technique with 2000 repeated samples [84]. The 95% confidence interval was tested by the bootstrap method of the bias-corrected percentile. The confidence interval does not contain a zero, indicating a significant direct effect. The direct effects between the examined variables can be seen in Figure 2 and Table 5. 

WLB had a negative association with EE (*β* = −0.75, *p* < 0.001), and the confidence interval (−0.79, −0.70) did not contain a 0, indicating that WLB had a negative direct effect on EE. The results supported hypothesis 1 that WLB is negatively correlated with EE.

EE had a negative association with WB (*β* = −0.38, *p* < 0.001), and the confidence interval (−0.47, −0.27) did not contain a 0, indicating that EE had a negative direct effect on WB. Therefore, the results supported hypothesis 2 that EE was negatively correlated with WB.

WLB had a positive association with WB (*β =* 0.51, *p* < 0.001), and the confidence interval (0.41, 0.60) did not contain a 0, indicating that WLB had a positive direct effect on WB. Thus, the results supported hypothesis 3 that WLB was positively correlated with WB. 

*R^2^* and ƒ^2^ were used to illustrate the effect value in this study. The values of *R*^2^ represent the compounding effects of the exogenous latent variables on the endogenous ones. According to Hair et al. [87], the *R^2^* value of 0.75 indicates a significant coefficient of determination, 0.50 a moderate one, and 0.25 a weak one. The compounding effect of WLB on EE was 56%, indicating a moderate effect value; the compounding effects of WLB and EE on WB were 69%, which was close to the significant effect value. Effect size ƒ^2^ is calculated by the formula ƒ^2^ = *R*^2^/(1 − *R*^2^). Effect size ƒ^2^ of 0.02 indicates a small effect of an exogenous latent variable, 0.15 a medium one, and 0.35 a significant one, respectively [88]. The effect size ƒ^2^ of WLB on EE was 1.27, indicating a significant effect of WLB on EE. The effect size ƒ^2^ of WLB and EE on WB was 2.23, indicating significant effects of these two exogenous latent variables on WB.

### 4.4. Indirect Effects Analysis

The bootstrapping technique was employed to analyze the indirect effect of WLB on WB through EE with 2000 repeated samples [84]. The results showed that the indirect effect of WLB on WB through EE was significant (β = 0.28, *p* < 0.001). The confidence interval (0.21, 0.36) did not contain a 0, indicating that WLB through EE had a significant indirect effect on well-being among Chinese college teachers. After controlling EE, the direct impact of WLB on WB was still significant, as shown in Table 5. The results revealed that EE played a partially mediating role in the relationship between WLB and WB among Chinese college teachers, which supported hypothesis 4.

## 5. Discussion

### 5.1. Characteristics of the Examined Variables among Chinese College Teachers 

#### 5.1.1. Gender Differences

This study revealed that gender was a significant factor linked to the WLB, EE, and WB of college teachers. Specifically, male college teachers in China had significantly weaker perceptions of WLB and WB than female teachers, while they had significantly stronger perceptions of EE than female teachers. Some Chinese scholars found that men experience more work overload [89], and their work pressure is much higher than that of women [90,91]. Nevertheless, previous studies on gender differences in WLB, EE, and WB have been inconsistent. For example, Vithanage and Arachchige found that female teachers scored slightly higher on WLB than male teachers [41], while other studies showed that male teachers had better WLB than female teachers [40]. Some studies found that female college teachers had higher WB scores than male teachers [44,45], while other studies found that college teachers’ perceptions of WB showed no significant gender difference [46]. In addition, some studies have found no gender difference in the EE scores of college teachers [48], while another study found that male teachers in local universities had stronger perceptions of EE than female teachers [91]. The results of this study provide further insights into gender differences in WLB, EE, and WB in a Chinese sample of college teachers. 

#### 5.1.2. Differences in College Types

This study found that the type of college (public vs. private) was also one of the factors affecting college teachers’ WLB, EE, and WB. To be specific, public college teachers had significantly weaker perceptions of WLB and WB than private college teachers, and they had significantly stronger perceptions of EE than private college teachers. Previous studies have revealed mixed results. Berheide et al. found that no significant difference in WLB scores existed between college teachers of public and private colleges [38]. Dinibutun et al. found that the EE perception of teachers in private colleges was stronger [49]. Akhtar and Saleem found that the WB of teachers in public universities was better than that of teachers in private universities [46]. It can be seen that the research results are different as a result of different research samples in different countries. This study investigated public and private college teachers in China and revealed the differences in the perceptions of the examined variables for that specific group of teachers.

#### 5.1.3. Differences in Roles of College Teachers

Significant differences existed in the WLB, EE, and WB of Chinese college teachers who are serving as class tutors and those who are not. To be specific, the perceptions of WLB and WB of college teachers who are serving as class tutors were significantly weaker than those of non-class tutors, and their perceptions of EE were significantly stronger than those of non-class tutors. Fang made a similar finding that those Chinese college teachers who concurrently serve as class tutors have significantly higher levels of EE perception than other college teachers [53]. Class tutors in Chinese colleges are responsible for the education and management of students [92]. They should guide students to study and perform self-management, and carry out mental health education [51]; therefore, they may face more responsibilities and difficulties [52]. This study is the first to explore the differences in the perceptions of the examined variables among college teachers who concurrently serve as class tutors or not, which fills the research gap in this field to some extent.

### 5.2. Relationships among WLB, EE, and WB

#### 5.2.1. WLB and WB

This study found that the WLB of Chinese college teachers was positively correlated with their WB. For college teachers, gender inequality, work pressure, and lack of a healthy workplace will lead to work-life imbalance, which negatively affects their WB [24]. The overlap of work and life domains, time pressure, reduction in leisure and free time, emotional reactions and cumulative work demands of college teachers all lead to work-life imbalance, which may affect their WB [93]. The COVID-19 pandemic has changed people’s work roles, increased their workload and led to work-life imbalance, which in turn affects people’s WB [94,95]. In contrast, WLB strengthens the positive correlation between good teacher-student relationships and WB [96]. This study provided further insights into Chinese college teachers’ WB in relation to WLB, especially in the post-COVID-19 era.

#### 5.2.2. WLB and EE

This study revealed that the Chinese college teachers’ WLB was negatively correlated with their EE. Studies have shown that overwork and role ambiguity often lead to work-life imbalance, which is positively correlated with EE [97,98]. Work demands also lead to work-life imbalance, which in turn leads to EE [99]. In particular, work-life imbalance caused by low income and long working hours is the main cause of EE [100]. During pandemic lockdowns, those with the worst WLB had higher EE [101]. These findings were similar to those of previous studies. However, few studies have focused on the correlation between WLB and EE among college teachers, and our results may help fill this research gap.

#### 5.2.3. EE and WB

This study revealed that the Chinese college teachers’ EE was negatively correlated with their WB, which is consistent with the results of Suárez Martel and Santana’s study [71]. College teachers are faced with job demands including teaching requirements, conflicts between teaching and research, and new challenges, which lead to EE, thereby reducing their job satisfaction and resulting in low WB [102]. Salas-Vallina et al. suggested that the absence of EE is a dimension of employee WB [69]. Lucas-Mangas et al. argued that EE is significantly negatively correlated with WB and factors related to WB [103]. If the work environment can reduce employees’ EE, it can increase their WB and job satisfaction [104]. Despite these studies on the correlation between EE and WB, few studies have investigated the relationship between the two variables among college teachers, and the present study provides empirical results for research in this field.

#### 5.2.4. The Mediating Role of EE

This study found that EE partially mediated the relationship between WLB and WB among Chinese college teachers, which supported the JD-R model-based hypothesis that job resources (WLB) promote positive work outcomes (WB) by reducing job burnout (EE) [74]. Employees who reported high EE were more likely to experience work-life imbalance and suffer low levels of WB [72]. For college teachers, job demands [97,98], especially work overload [99] and long working hours [100] will not only lead to work-life imbalance, but also lead to EE, and both of them together result in low WB [102]. As far as we know, this study was the first to examine EE as a mediator between WLB and WB.

## 6. Conclusions and Suggestions

### 6.1. Conclusions

Male teachers, college class tutors, and public college teachers had lower scores on WLB and WB, but higher scores on EE than female teachers, non-class tutors, and private college teachers, respectively. In conclusion, the results of this study provide insights into differences in WLB, EE, and WB among college teachers of different genders and college types. 

Among Chinese college teachers, WLB had a negative relationship with EE, but a positive relationship with WB, and EE had a negative relationship with WB. In addition, this study showed that EE partially mediated the association between WLB and WB. These findings indicate that WLB plays an important part in reducing EE and enhancing WB for Chinese college teachers.

### 6.2. Contributions

This study explored well-being in relation to work-life balance (WLB) and emotional exhaustion among Chinese college teachers, and examined emotional exhaustion as a mediator between WLB and well-being for the first time, which helps fill the research gap in this respect. 

This study provides empirical evidence for the JD-R theory with a sample of college teachers in China. In addition, this study explored for the first time the differences in the perceptions of WLB, EE, and WB among college teachers who concurrently serve as class tutors or not, thus filling the research gap in this field to some extent. It also helps to explore ways to alleviate the stress of work-life imbalance and reduce the risk of emotional exhaustion, and it provides references for exploring sustainable development paths for college teachers’ well-being.

### 6.3. Application Suggestions

Excessive workload [89] and high work pressure [90,91] are the main factors resulting in weak perceptions of WLB and WB, and strong perceptions of EE among male teachers in Chinese colleges. Although the perceptions of WLB and WB of female college teachers in China were stronger than those of male teachers, the result is still not optimistic as it showed that Chinese female college teachers’ perceptions of WLB, WB, and EE were higher than the median value, which is also worthy of attention. Therefore, it is suggested that universities should pay attention to the workload of teachers and plan for appropriate work tasks and time [30]. If excessive workload cannot be reduced, teachers in colleges can be trained to adopt an evasive coping style, which can reduce the impact of excessive workload on their EE [30].

Role ambiguity, that is, the overlap of responsibilities among class tutors, full-time teachers, and counselors, is a main factor which causes work pressure for college teachers serving as class tutors in China [52]. It is suggested that colleges should clarify the responsibilities among different roles of college teachers to reduce the work pressure of college teachers serving as class tutors, which may help improve their WLB, reduce their EE, and increase their WB.

Although the perceptions of WLB and WB of teachers in private colleges were stronger than those of teachers in public colleges, and their perceptions of EE were weaker than those of teachers in public colleges, private college teachers’ perceptions of EE in China were still higher than the median value and thus deserve attention. Both public and private college teachers in China are under pressure from teaching, research [105], and promotion for professional titles [106]. Therefore, it is suggested that colleges should assign appropriate teaching workloads and establish a reasonable promotion mechanism for professional titles, as well as a scientific assessment mechanism for research [105]. Colleges should provide psychological assistance for teachers faced with excessive pressure, because psychological assistance programs can significantly reduce work pressure [107].

As WLB helps to reduce EE and promote WB, it is suggested that measures should be taken to increase the WLB of college teachers. It may be useful to arrange appropriate workloads, provide necessary services and support for scientific research and professional title promotion for college teachers, and develop WLB practices such as flexible working hours, telecommuting, job sharing, and childcare services [108]. 

In addition, EE can weaken the positive relationship between WLB and WB, so there is a need for prevention and intervention practices of EE. As excessive workload [109] and excessive stress [47,110] are the main factors leading to the EE of college teachers, it is suggested that colleges should take measures to reduce excessive workload and enhance teachers’ sense of work meaning, which may also reduce the exhaustion caused by stress [111]. For example, WB-oriented management would be helpful [112].

### 6.4. Limitations and Suggestions for Future Study

Firstly, this study only explored college teachers’ WB in relation to their WLB and EE, which revealed the mediating effect of EE on WLB and WB. However, there may be other influencing factors related to WB, so future research could focus on other variables such as perceived organizational support. Studies have shown that perceived organizational support is a positive factor linked to employees’ WB [113] and their creativity [114], which is one of the foundations of teachers’ WB. In addition, recent studies have shown that general factors of character strength can significantly explain differences in WB among people. Therefore, studies on college teachers’ WLB, perceived organizational support, character strengths, and WB deserve attention in the future.

Secondly, the results of this study supported the hypotheses that WLB is positively correlated with WB, but negatively correlated with EE. However, there are many factors associated with college teachers’ WLB, EE, and WB. Qualitative research methods could be used in the future to explore the specific factors influencing college teachers’ WLB, EE, and WB.

Thirdly, although China has entered a post-epidemic era, the risk of the epidemic still exists and people’s work and lives are still affected by it. Therefore, future studies may focus on the influence of fear and anxiety about the epidemic and that of epidemic prevention and control on the WLB of college teachers, and explore the impacts of these pandemic-related factors on EE and WB through their impact on WLB. 

Fourthly, since snowball sampling is a non-probability sampling technique which cannot guarantee the representativeness of the population, other sampling methods should be combined to ensure the representativeness of future samples. The snowball sampling method cannot balance the equilibrium of demographic variables, such as gender, which will inevitably increase gender bias. The gender bias could be overcome in future studies by balancing the gender of the subjects.

## Figures and Tables

**Table 1:** CFA Fit Indexes and Discriminant Validity.

Index	χ^2^/*df*	RMSEA	GFI	AGFI	*t*
**Threshold**	**<5**	**<0.10**	**>0.80**	**>0.80**	**>3**
**Work-life balance**	3.65	0.07	0.99	0.96	22.57~26.58
**Emotional Exhaustion**	2.89	0.06	0.99	0.97	19.01~23.22
**Well-being**	5.51	0.08	0.93	0.90	22.85~28.52

**Table 2:** Reliability and validity scores for each variable.

Variables	α	CR	AVE	FL	M ± SD	Max
**Criteria**	>0.70	>0.70	>0.50	>0.50	-	-
**Work-life balance**	0.92	0.91	0.69	0.70~0.88	3.10 ± 0.95	5
**Emotional Exhaustion**	0.90	0.90	0.66	0.79~0.92	3.60 ± 1.19	5
**Well-being**	0.87	0.93	0.58	0.51~0.86	3.15 ± 1.00	5

**Table 3:** Discriminant Validity Analysis.

Construct	Work-Life Balance	Emotional Exhaustion	Well-Being
**Work-life balance**	**0.83**		
**Emotional Exhaustion**	−0.69	**0.81**	
**Well-being**	0.74	−0.72	**0.76**

Note: The bold value indicates the square root of the AVE for each dimension.

**Table 4:** Differences Analysis of College Teachers’ WLB, EE, and WB.

	WLB	EE	WB
**Gender**			
**Male**	2.96 ± 0.99	3.79 ± 1.19	3.04 ± 1.04
**Female**	3.19 ± 0.91	3.48 ± 1.17	3.22 ± 0.98
***t***	−2.76 **	2.99 **	−2.13 *
Class tutors			
**yes**	2.91 ± 0.92	3.90 ± 1.09	2.93 ± 0.96
**no**	3.48 ± 0.86	3.02 ± 1.15	3.58 ± 0.95
***t***	−7.33 ***	9.18 ***	−7.93 ***
College types			
**public**	3.04 ± 0.95	3.71 ± 1.18	3.01 ± 0.96
**Private**	3.20 ± 0.92	3.41 ± 1.18	3.31 ± 1.04
***t***	−2.01 *	2.89 **	−3.04 **

Note: * represents *p* < 0.05, ** represents *p* < 0.01, *** represents *p* < 0.001.

**Table 5:** Direct and Indirect Effects.

Parameter	Estimate	Lower Bounds	Lower Bounds
Standard direct effects			
WLB → EE	−0.75 ***	−0.79	−0.70
WLB → WB	0.51 ***	0.41	0.60
EE → WB	−0.38 ***	−0.47	−0.27
Standard indirect effects			
WLB → EE → WB	0.28 ***	0.21	0.36

Note: *** The empirical 95% confidence interval does not contain zero. *** *p* < 0.001.

## Data Availability

The original contributions presented in the study are included in the article; further inquiries can be directed to the corresponding author.
